# The autoregulatory serglycin/CD44 axis drives stemness‐like phenotypes in TNBC in a β‐catenin‐dependent manner

**DOI:** 10.1002/ctm2.311

**Published:** 2021-02-01

**Authors:** Li Cao, Fei‐Fei Luo, Hong‐Bin Huang, Tie‐Jun Huang, Hao Hu, Li‐Sheng Zheng, Jing Wang, Li‐Xia Peng, Chao‐Nan Qian, Bi‐Jun Huang

**Affiliations:** ^1^ State Key Laboratory of Oncology in South China and Collaborative Innovation Center for Cancer Medicine Sun Yat‐sen University Cancer Center Guangzhou People's Republic of China; ^2^ School of Pharmaceutical Science Sun Yat‐sen University Guangzhou People's Republic of China; ^3^ Department of Pharmacy Sun Yat‐Sen University Cancer Center Guangzhou People's Republic of China; ^4^ Department of Nuclear Medicine The Second People's Hospital of Shenzhen Shenzhen People's Republic of China; ^5^ Department of Traditional Chinese Medicine The First Affiliated Hospital of Sun Yat‐Sen University Guangzhou People's Republic of China; ^6^ Department of Nasopharyngeal Carcinoma Sun Yat‐Sen University Cancer Center Guangzhou People's Republic of China; ^7^ Department of Radiation Oncology Guangzhou Concord Cancer Center Guangzhou China

Dear Editor,

Overexpression of serglycin (SRGN) in human breast cancer suggested a poor prognosis. However, its role in triple‐negative breast cancer (TNBC) recurrence and metastasis remains unclear.^1^, ^2^, ^3^ To explore the clinical value of SRGN, we used 144 breast cancer samples to determine SRGN expression (Figures 1A and 1C). The SRGN expression level was closely associated with the overall survival of breast cancer (Figure 1B; Figure S1A). Additionally, immunohistochemistry (IHC) staining showed that serglycin was expressed in the tumor stroma (Figure 1F). EGFR and Ki67, which were significantly correlated with worse survival and a poor prognosis in TNBC patients, showed higher expression levels in TNBC tissue.^4^ The luminal subtype expressed GATA3, which regulates differentiation and suppresses dissemination in breast cancer.^5^ We found that SRGN was positively correlated with EGFR and Ki67. Additionally, we found that the SRGN mRNA level was negatively correlated with GATA3 (Figures 1G, 1L, and 1M; Figures S1B‐S1D).

**FIGURE 1:**
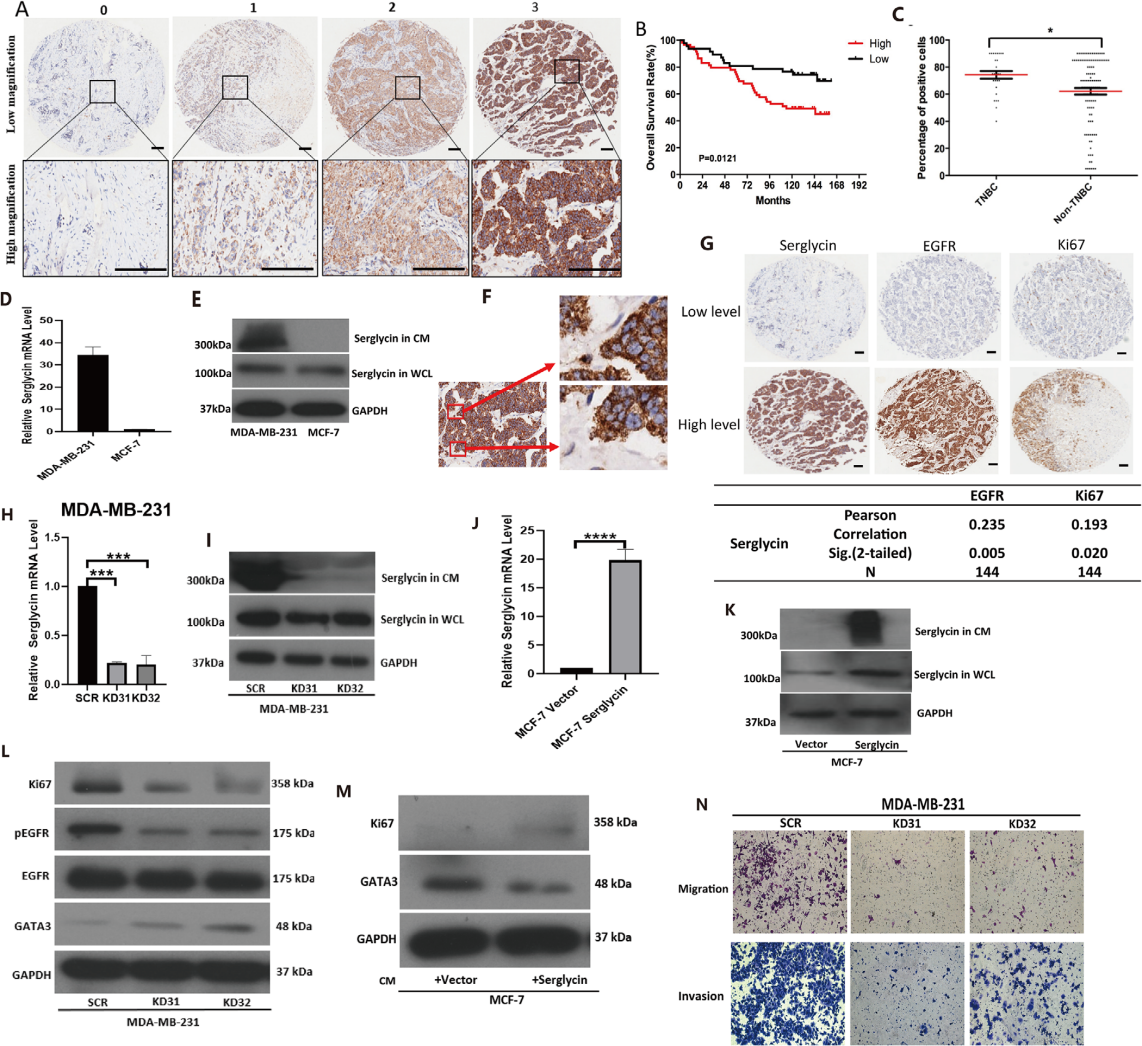
(A) Immunohistochemistry (IHC) of SG was performed in 144 human breast cancer. Different intensity of SG staining (0, 1, 2, 3, 4) are shown under both low and high magnifications of a light microscope. Scale bars 100 µm.(B) Kaplan‐Meier survival curve shows the overall survival of patients displaying low (<70%) and high (>70%) percentage of stained cells, and the patient numbers of low and high are 66 and 78, respectively. (C) The difference of SG positive cells’ percentage between triple negative breast cancer (TNBC) patients (27) and Non‐TNBC patients (117) is shown. (D) The mRNA level of SG (normalized to GAPDH) in two cell lines (MDA‐MB‐231, MCF‐7) was confirmed by quantitative real‐time PCR (from triplicates). (E) Cultured MDA‐MB‐231 and MCF‐7 cells in serum‐free medium for 48 hours, collected culture medium (CM) and whole cell lysate (WCL) to Western blotting assay, the SG protein level in these two cell lines were determined. (F) SG expressed in TNBC, the right side shows high magnification of two sites. Speculate margins outside the cells represent SG secretion in tissue. (G) IHC of SG, EGFR, Ki67 were performed in 144 human breast cancer, low and high percentage of stained cells are showed. Scale bars 100 µm. Association of SG and EGFR or Ki67 respectively in 144 breast cancer patients by IHC in the table. (H) The mRNA level of SG (normalized to GAPDH) in knockdown cells was confirmed by quantitative real‐time PCR (from triplicates). (I) The SG protein level of knockdown cells was determined by Western blotting, suppression of SG in KD31 and KD32 cells dramatically eliminated the secretion of SG in CM. (J) The mRNA level and protein level of SG in overexpression cells were examined (from triplicates). (K) The SG protein level in both CM and WCL were increased. (L) Western blotting assay to examined protein level change of Ki67, EGFR, GATA3 when Knocked down SG expression. (E‐2) SRGN mRNA level is negatively correlated to GATA3, p < 0.0001. (M) Western blotting assay to examined protein level change of Ki67, GATA3 when overexpressed SG. (N) Stably transfected cells were subjected to migration and invasion assay, cells crossed the membrane were fixed with methanol, followed by crystal violet staining, image was taken by the microscope (100x)

To reconfirm the findings above, we examined SRGN expression in the cell lines of MDA‐MB‐231 and MCF‐7, which are representative cell lines of the basal‐like and luminal subtypes, respectively. The results showed that serglycin in MDA‐MB‐231 was significantly higher either at the mRNA level or protein level compared with MCF‐7 (Figures 1D and 1E; Figure S1E).

Here, we showed that the migration and invasion and wound healing of TNBC cells could be remarkably suppressed in vitro by stably knocking down SRGN (Figures 1H‐1K and 1N; Figures 2A‐2C; Figure S1F). In vivo, the results showed a drastically lower rate of lung metastasis in mice injected with the SRGN knockdown cell lines (MDA‐MB‐231‐KD31 and KD32) than in those with MDA‐MB‐231‐SCR cells (Figures 2D‐2G). The stemness of TNBC, such as the self‐renewal and tumor‐initiating capacities of breast cancer cells, was also mediated by SRGN (Figures 2H‐2L; Figures S1G and S1H). Western blotting showed that KD32 cells highly expressed cleaved‐PARP (Figure 3A), suggesting that SRGN promoted TNBC cells to avoid anoikis. Remarkably, SRGN expression is related to the tumor‐initiating capacity in vivo, and the xenograft MDA‐MB‐231 KD32 showed the better survival than the SCR in mice (Figures 3B and 3C; Figure S2A). In contrary, we used MCF‐7 cells to generate stable overexpression cell lines, and we showed that SRGN overexpression in MCF‐7 cells was closely associated with conversion between luminal and basal‐like properties (Figures 1J, 1K and 1M). In addition, downregulation of SRGN promotes the sensitivity to chemotherapy (Cisplatin and Doxorubicin) in MDA‐MB‐231 cells rather MCF‐7 cells (Figures 3D‐3I; Figures S2B‐S2E).

**FIGURE 2:**
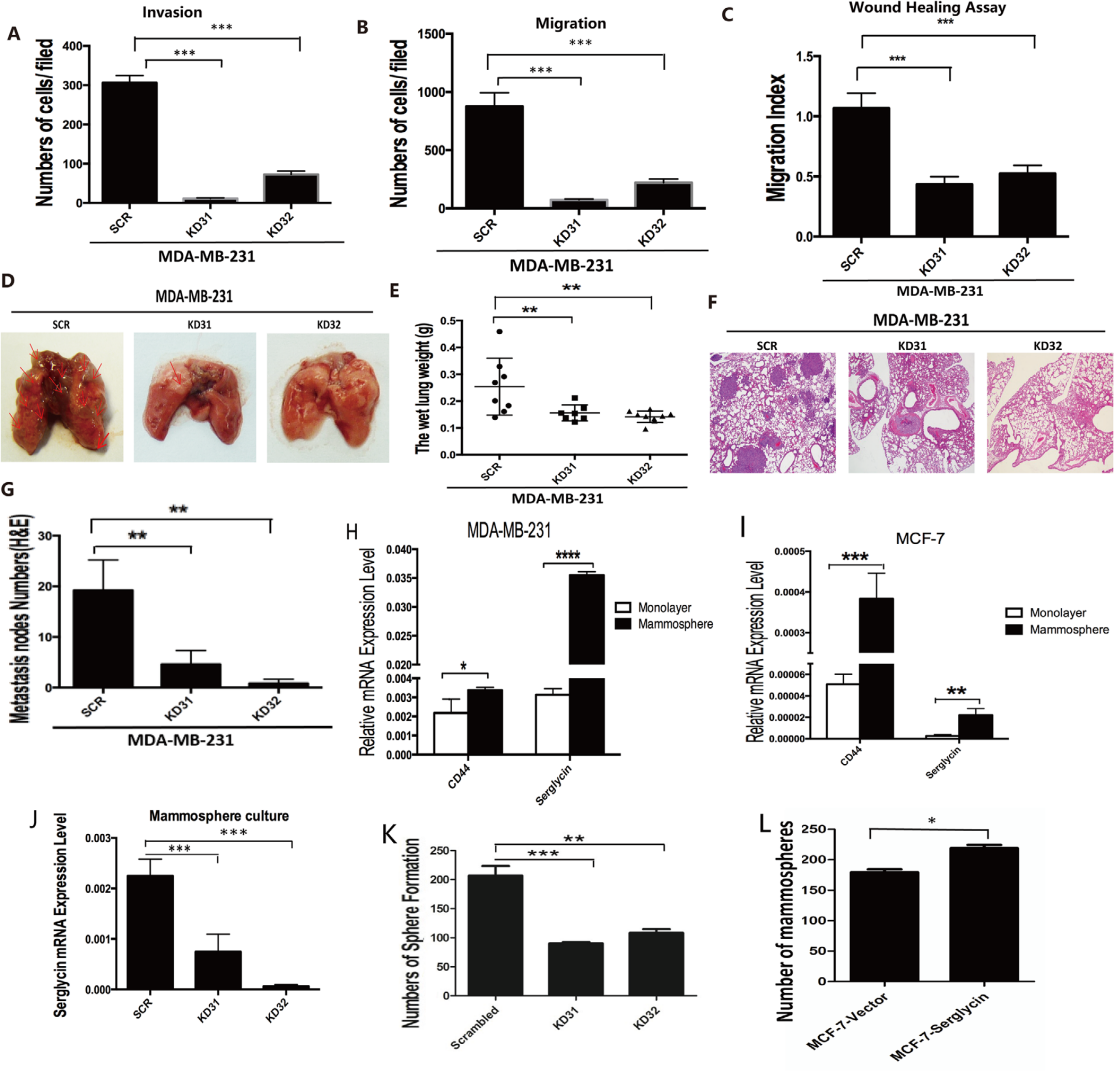
(A) Stably transfected cells were subjected to invasion assay, the numbers of crossed cells counted by ImageJ are shown (from triplicates). (B) Stably transfected cells were subjected to migration assay, the numbers of crossed cells counted by ImageJ are shown (from triplicates). (C) Histogram shows the migration index of each kind of cells in wound healing assay. (D) Stably transfected cells (SCR, KD31, KD32) were injected in mice via tail vein, sacrificed the mice 8 weeks later, the lung of mice was washed by 0.9% Nacl. Representative images were shown. (E) The wet lung weight was recorded. (F) Lung sections were stained with hematoxylin and eosin. (G) The numbers of microscopic metastatic nodules in the sections were counted. (H and I) Culture MDA‐MB‐231 and MCF‐7 cells in normal way and in mammosphere way, total RNA was extracted from all the cells. Subjected these to the quantitative real‐time PCR (from triplicates) to determined mRNA level change of CD44 and SG (normalized to GAPDH). (J) Spheres’ mRNA level of SG (normalized to GAPDH) was confirmed by quantitative real‐time PCR (from triplicates). (K and L) The numbers of spheres generated by MDA‐MB‐231 and MCF‐7 cells were shown. Data were presented as the mean ± SD of three independent experiments. *p < 0.05, **p < 0.01, ***p < 0.001 by student's *t*‐test

**FIGURE 3:**
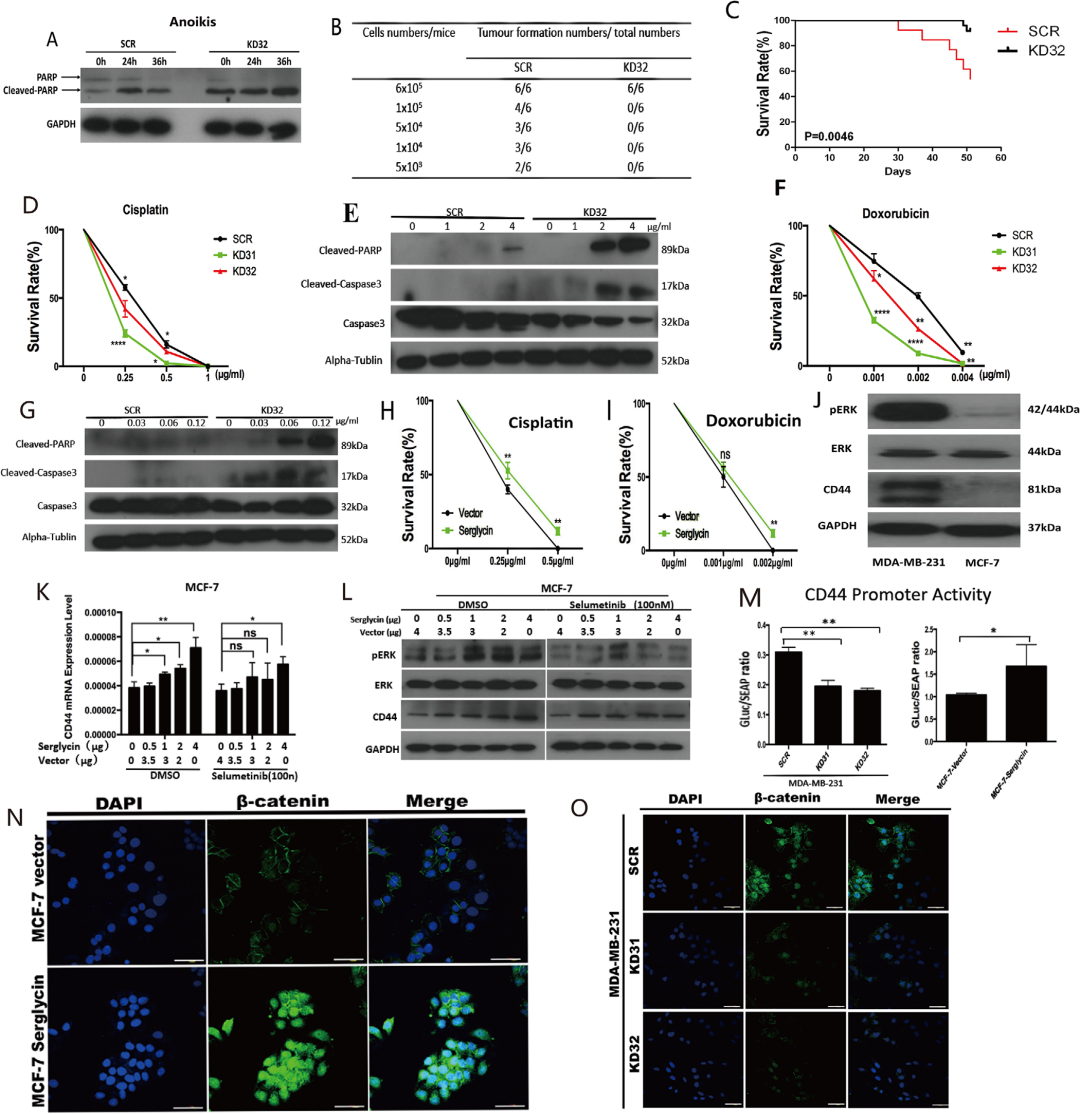
(A) Cells (1 × 10^5^/well) were culture on six‐well ultra‐low attachment plates using serum free medium for 24 hours, 36 hours. Collected total protein to examined expression of cleaved‐PARP by Western blotting. (B) Tumor formation in nude mice when SCR or KD32 cells were injected subcutaneously, results of tumor formation numbers in different groups were shown. (C) Survival curve of orthotopic tumour implantation nude mice was shown. (D and F) The survival rate of cells treated with Cisplatin or Doxorubicin was shown. KD31 or KD32 was compared with SCR, respectively. (E and G) The expression of apoptosis marker in SCR and KD32 treated with Cisplatin or Doxorubicin was detected by Western blotting. (H and I) The survival rate of cells treated with Cisplatin or Doxorubicin were shown. MCF‐7‐serglycin was compared with MCF‐7 vector. (J) The protein level of CD44 and p‐ERK in two wild type cells were analyzed by Western blotting. (K) MCF‐7 cells treated with Selumetinib were transiently transfected with serglycin plasmid. The mRNA level of CD44 was determined by quantitative real‐time PCR. (L) The protein level of CD44 and p‐ERK was analyzed by Western blotting. (M) CD44‐luciferase activity of stably knockdown cells (left) or stably overexpression cells (right) was analyzed. (N and O) The expression level and the localization change of β‐catenin in stably knockdown cells (SCR, KD31, KD32) (O) and stably overexpression cells (MCF‐7‐Vector, MCF‐7‐serglycin) (N) were confirmed by confocal immunofluorescence, scale bars 50 µm. Data were presented as the mean ±SD of three independent experiments. *p < 0.05, **p < 0.01, ***p < 0.001 by student's *t*‐test

The aggressive phenotype of TNBC might be partially due to an abundance of cancer stem cells (CSCs), indicating that TNBC has more CSC‐like properties.^6^ Therefore, we examined the expression of the CSC marker CD44.^7^, ^8^ Real‐time quantitative PCR (qPCR) showed that cells in mammospheres generated by MDA‐MB‐231 and MCF‐7 cells expressed a high CD44 level, indicating that mammospheres can enrich CSCs. Thus, CD44 might be a CSC marker for breast cancer and serves as a receptor of serglycin. We found the same with CD44, other CSC markers such as Nanog, ALDH1, and CD133 high expression in MDA‐MB‐132 than MCF‐7. These CSC markers significantly reduced in the silencing of SRGN in MDA‐MB‐231, but we could not see significantly change in MCF‐7 over expression serglycin cells (Figures S2F, S2H, and S2I;)

We examined the expression of CD44 and phosphor‐ERK (P‐ERK) in two wild‐type cell lines. We found that CD44 and P‐ERK were both highly expressed in MDA‐MB‐231 cells (Figure 3J; Figure S2G), indicating that MAPK signaling was overactivated in MDA‐MB‐231 cells, and SRGN, CD44, and P‐ERK may interact with each other. These data indicate that the expression of SRGN was positively correlated with CD44 and can promote the phosphorylation of ERK to activate MAPK signaling.

To verify the connection between P‐ERK and CD44 in TNBC, we used different concentrations of the specific ERK inhibitor selumetinib or U0126 to treat two wild‐type cells and found that P‐ERK was effectively suppressed, accompanied by decreased CD44 expression in both cell lines (Figures 3K and 3L; Figures S3A and S3B). However, when the MFC‐7 cells were treated with U0126 or selumetinib, both CD44 and P‐ERK did not significantly increase (Figures S3I and S3J). Additionally, under the treatment of different concentrations of selumetinib and U0126, the numbers of mammospheres formed by MDA‐MB‐231 cells were markedly decreased, while they in MCF‐7 did not show a significant change (Figures S3C‐S3H). To investigate how CD44 expression is mediated, we performed the luciferase report assay using a CD44 promoter construct (Figure 3M). These results revealed that CD44 expression was regulated by a serglycin‐activating pathway at the transcriptional level.

Moreover, we demonstrated serglycin promoted nucleus translocation of β‐catenin and the β‐catenin expression level in two wild‐type cells were evaluated by immunofluorescence assay (Figures 3N and 3O; Figure S4A) and Western blotting (Figures 4B and S4C). Serglycin was directly binding with CD44 on the cytomembrane of MDA‐MB‐231(Figures 4A and 4B; Figure S4D). We found the binding of SRGN to CD44 can be prevented by a CD44‐neutralizing antibody in MDA‐MB‐231 cells (Figure 4C). The CD44‐neutralizing antibody could significantly reduce the migration and the formation of mammospheres in a dose‐dependent manner by blocking the binding of SRGN to CD44 (Figures S4E‐S4G). Thus, we demonstrated that combined treatment with DDP and the CD44‐neutralizing antibody suppressed the cell proliferation better than single drug treatment separately (Figures 4D‐4F). The effect from binding SRGN with CD44 can be restrained by CD44 neutralizing antibody in a β‐catenin‐dependent manner (Figure 4G).

**FIGURE 4:**
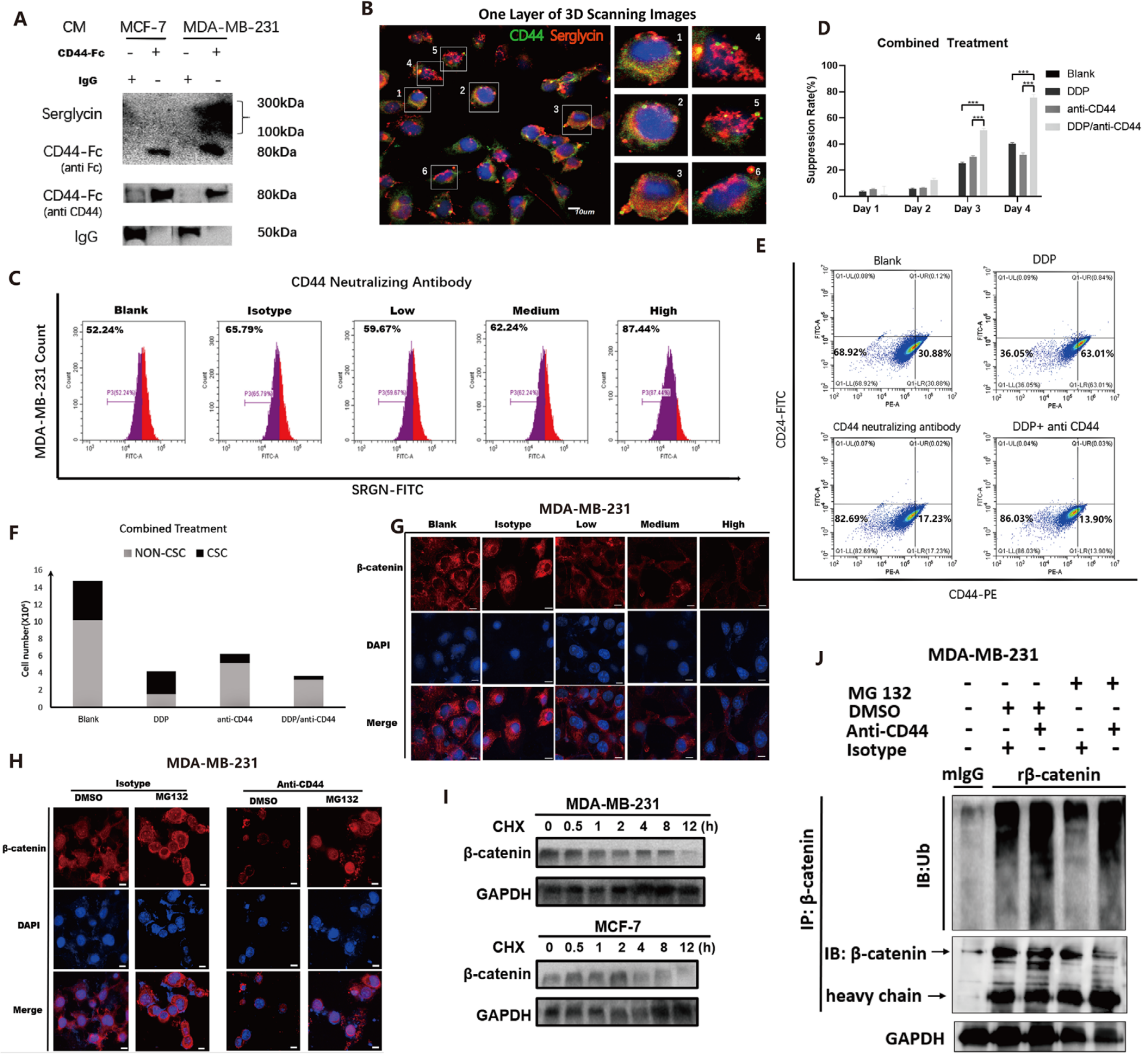
(A) Serglycin was precipitated with CD44‐Fc from the conditioned medium from MDA‐MB‐231 cells. (B) Immunofluorescence assay had also shown that SRGN and CD44 were colocalization on the membrane of MDA‐MB‐231, scale bars 10 μm. (C) The FCM assay showed this binding between SRGN and CD44 can be prevented by CD44 neutralizing antibody in MDA‐MB‐231 cells in dose‐dependent. (D) MTS assay was conducted to demonstrate the suppression effects on the proliferation of MDA‐MB‐231 cells, and combined treatment can better suppress the cells proliferation than separately treatment for 3 days. (E) The FCM assay showed combined DDP and CD44 neutralizing antibody treatment is preferably than separately usage, and CD44 neutralizing antibody could target CSC. (F) Bar graph showed the cell number of CSC and non‐CSC after combined DDP and CD44 neutralizing antibody treatment through FCM assay. (G) The expression level and the localization of β‐catenin in MDA‐MB‐231 after being treated with different concentration CD44 neutralizing antibody as before were confirmed by confocal immunofluorescence, scale bars 10μm. (H) The expression level of β‐catenin could be degraded by high concentration CD44 neutralizing antibody in dose‐dependent. This degradation could be reversed by MG132 which means it is dependent on the ubiquitination degradation ways. (I) MDA‐MB‐231 cells and MCF‐7 cells were treated with cycloheximide (CHX; 20 μg/mL) for the indicated periods of time. β‐catenin levels were analyzed by immunoblotting. (J) MDA‐MB‐231 cells were treated with MG132 (5 μM) or DMSO or treated with CD44 neutralizing antibody (1 μg/mL) or isotype for 24 hours. Cell lysates were immunoprecipitated (IP) with either control IgG or antibody against β‐catenin and analyzed by immunoblotting with a ubiquitin (Ub)‐specific antibody. Bottom, GAPDH from input cell lysates

Consequently, the expression level of β‐catenin could be degraded by a high concentration of the CD44‐neutralizing antibody in a dose‐dependent manner. This ubiquitination degradation of β‐catenin in MDA‐MB‐231 cells could be reversed by the proteasome inhibitor MG132 (Figures 4H‐4J). These results indicated that the extracellular matrix (ECM) factor SRGN binds to cell surface‐adherent CD44 in an autocrine manner, resulting in activation of the MAPK signaling pathway to trigger the translocation of β‐catenin into the nucleus, which, in turn, regulates CD44 expression. The CD44‐neutralizing antibody competitively inhibits SRGN binding with CD44 and suppresses the MAPK pathway depending on β‐catenin ubiquitination degradation pathways. Thus, this result sheds light on breast CSC treatment targeting CD44 (Figure S4H).

## CONFLICT OF INTEREST

The authors declare that there is no conflict of interest that could be perceived as prejudicing the impartiality of the research reported.

## ETHICS APPROVAL AND CONSENT TO PARTICIPATE

All protocols with human specimens were applied under the examination and approval of the Ethical Committee of Sun Yat‐Sen University Cancer Center.

### AUTHOR CONTRIBUTIONS

Fei‐Fei Luo and Li Cao conducted the in vitro and in vivo experiment and data analysis and wrote the manuscript. Hong‐Bin Huang, Tie‐Jun Huang, Li‐Xia Peng, and Li‐Sheng Zheng collected the patient samples and the follow‐up information and performed the clinical data analysis. Hao Hu and Jing Wang performed the bioinformatics analysis. Chao‐Nan Qian and Bi‐Jun Huang designed the study and analyzed the manuscript. All the authors read and approved the final manuscript.

### DATA ACCESS, RESPONSIBILITY, AND ANALYSIS

All data generated or analyzed during the present study are available via the corresponding author on reasonable request. The data are under review and will link to Research Data Deposit (http://www.researchdata.org.cn/) with a unique deposit ID: RDDB2021001067.

### FUNDING INFORMATION

This work was supported by grants from the National Natural Science Foundation of China (grnat numbers: 81972785, 81773162, and 81572901 [Bi‐Jun Huang]; 81872384, 81672872, and 81472386 [Chao‐Nan Qian]), the Provincial Natural Science Foundation of Guangdong, China (grant numbers: 2017A030313866 [Bi‐Jun Huang], 2016A030311011 [Chao‐Nan Qian]) and the Three Key Research Programs of SYSU (grant numbers: 84000–18843409 [Chao‐Nan Qian]).

## Supporting information

Supporting Information

Supporting Information

Figure S1 (A‐E) Based on the bc‐GenExMiner v4.1 databases. (A) SRGN low expression had a significantly better lung metastasis free survival rate than that of high‐expression patients HR = 1.68(1.13, 2.5), p = 0.01087. (B) SRGN mRNA level is positively correlated to EGFR, p < 0.0001. (C) SRGN mRNA level is negatively correlated to GATA3, p < 0.0001. (D) SRGN mRNA level is correlated to KI67 but not statistically significant, p = 0.1201. (E) SRGN mRNA level is significantly increased in Basal‐like TNBC (n = 243), p < 0.0001. (F) Representative phase contrast images of wound healing assay are shown, the white lines denote the width of the wound. (G) Representative images of spheres generated by SCR, KD31, KD32 were shown, scale bars 50 µm. (H) Representative images of spheres generated by MCF‐7‐Vector and MCF‐7‐serglycin were shown, scale bars 50 µm

Figure S2 (A) Tumor formation in nude mice when SCR or KD32 cells were injected subcutaneously into right or left armpit. (B and C) Representative images of colonies generated by SCR, KD31, and KD32, treated with different concentration of Cisplatin or Doxorubicin were shown. (D and E) Representative images of colonies generated by MCF‐7‐Vector and MCF‐7‐serglycin, treated with different concentration of Cisplatin or Doxorubicin were shown. (F) SRGN mRNA level is positively correlated to CD44, p < 0.0001 based on the bc‐GenExMiner v4.1 databases. (G) The protein level of CD44 and p‐ERK in stably transfected cells were analyzed by Western Blotting. (H and I) CSC markers such as CD44, Nanog, ALDH1, and CD133 mRNA expression in MDA‐MB‐132 and MCF‐7 by quantitative real‐time PCR. Data were presented as the mean ± SD of three independent experiments. *p < 0.05, **p < 0.01, ***p < 0.001 by student's *t*‐test

Figure S3 (A and B) The protein level change of CD44 and p‐ERK in two wild type cells treated with different concentration of Selumentinib or U0126 (ERK inhibitor) was presented. (C and D) Representative images and bar graph of tumor spheres formed by two wild type cells, treated with increased dose of Selumetinib were shown, scale bars 50μm. (E and F) Representative images and bar graph of tumor spheres formed by two wild type cells, treated with increased dose of U0126 were shown, scale bars 50μm. (G and H) Numbers of mammospheres were counted after being treated with Selumetinib or U0126. (I and J) MCF‐7 cells treated with U0126 were transiently transfected with serglycin plasmid. The mRNA level of CD44 was determined by quantitative real‐time PCR (J). The protein level of CD44 and p‐ERK was analyzed by Western Blotting (I).

Figure S4 (A) The expression level and the localization of β‐catenin in MDA‐MB‐231 and MCF‐7 were confirmed by confocal immunofluorescence, scale bars 50 µm. (B and C) Nuclear (N) and cytosolic/membrane (C+M) protein from stably overexpression cells (B) or stably knockdown cells (C) were subjected to examined β‐catenin change by Western Blotting. LMNB1 and GAPDH were used as loading control of N and C+M, respectively. (D) Immunofluorescence assay had also shown that SRGN and CD44 were colocalization on the membrane of MDA‐MB‐231, scale bars 20μm. (E) The ability of migration and self‐renewal of MDA‐MB‐231 cells can be prevented by CD44 neutralizing antibody in dose‐dependent. (F) The migration numbers of crossed cells counted by ImageJ are shown (from triplicates). (G) Numbers of mammospheres which represented self‐renewal of MDA‐MB‐231 cells were counted. (H) The CD44 neutralizing antibody is competitive inhibiting SRGN binding with CD44, meanwhile. Ultimately, the CD44 neutralizing antibody could inhibit the EMT, self‐renewal, and chemoresistanse of MDA‐MB‐231 by the MAPK pathway.
