# Supplementary material for: The autoregulatory serglycin/CD44 axis drives stemness‐like phenotypes in TNBC in a β‐catenin‐dependent manner
Source: Clin Transl Med. 2021 Feb 1;11(2):e311. doi: 10.1002/ctm2.311 (PMC7851355; doi:10.1002/ctm2.311)
Supplement: Supplementary file 2 — Supporting Information [file CTM2-11-e311-s006.docx]

CD44 promoter sequence：

aaataaatactgcgtttgatttccaaacattaaaccatagtatattatagatagatatagagttatcattcaaagtatgatatttcaatctcaaaaggcttcccctgaagaatattacaaactcttcctctctttaagatctgctgggtaggaaagatgggagaaaatgaattaatgtttacacagaaaggaggataatgggggcaaaaataatagatgaacgtatgggtggatgagagaatggataaaatgataggtggatatgttgatcttggacagatgggaaatgagtggatatatcaataaacagatatgtgggtggatgggtggagaagaggatggtggatggttgtggttttatgaagagatgtgaaaaaggaagtgtggaatgatggatgagaagttgtatgggaagatgaatagaagaataggtggttgaataaattaaaaggtgtgtggttggatgaatgaatgagtgggatgatagatggacctaagtggttagtggatggacaggaggatggatggatgtgagagccccagaaggacataaggaaagatgggtggatagatggatgggcggatggaaggatatttaggaggatgaatgagcatgtgtgtggagagaggtgcccattcacactggcttgaacacatgggttagctgagccaaatgccagccctatgacaggccatcagtagctttccctgagctgttctgccaagaagctaaaattcattcaagccatgtggacttgttattgaggggaaaaagaatgagctctccctctttccacttggaagattcaccaactccccacccctcactccccactgtgggcacggaggcactgcgccacccagggcaagacctcgccctctctccagctcctctcccaggatatccaacatcctgtgaaacccagagatcttgctccagccggattcagagaaatttagcgggaaaggagaggccaaaggctgaacccaatggtgcaaggttttacggttcggtcatcctctgtcctgacgccgcggggccagcgggagaagaaagccagtgcgtctctgggcgcaggggccagtggggctcggaggcacaggcaccccgcgacactccaggttccccgacccacgtccctggcagccccgattatttacagcctcagcagagcacggggcgggggcagaggggcccgcccgggagggctgctacttcttaaaacctctgcgggctgcttagtcacagccccccttgcttgggtgtgtccttcgctcgc tccctccctccgtcttaggtcactgttttcaacctc
