# Supplementary figures and images for: The autoregulatory serglycin/CD44 axis drives stemness‐like phenotypes in TNBC in a β‐catenin‐dependent manner
Source: Clin Transl Med. 2021 Feb 1;11(2):e311. doi: 10.1002/ctm2.311 (PMC7851355; doi:10.1002/ctm2.311)

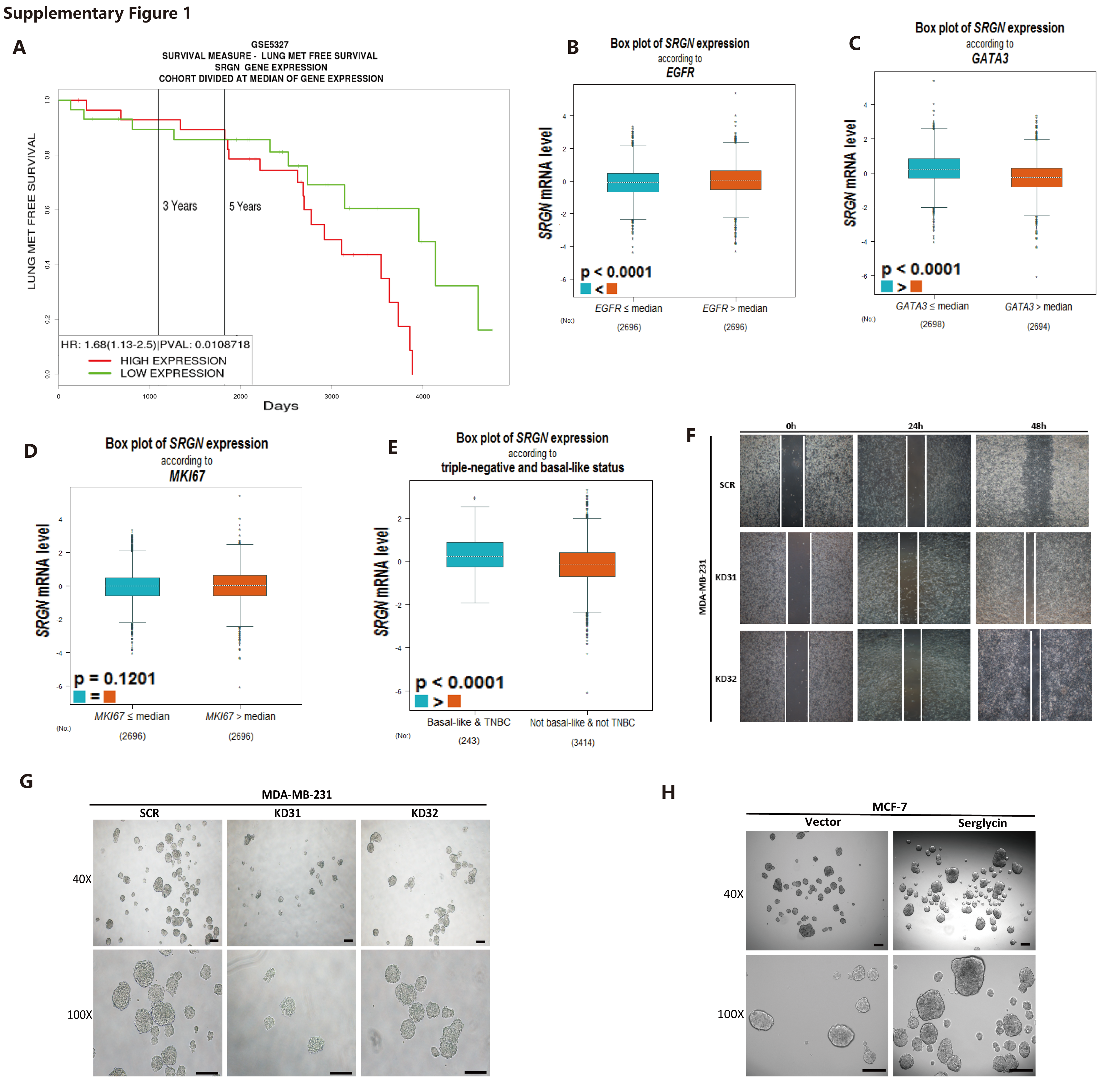

Supplement: Supplementary file 3 — Figure S1 (A‐E) Based on the bc‐GenExMiner v4.1 databases. (A) SRGN low expression had a significantly better lung metastasis free survival rate than that of high‐expression patients HR = 1.68(1.13, 2.5), p = 0.01087. (B) SRGN mRNA level is positively correlated to EGFR, p < 0.0001. (C) SRGN mRNA level is negatively correlated to GATA3, p < 0.0001. (D) SRGN mRNA level is correlated to KI67 but not statistically significant, p = 0.1201. (E) SRGN mRNA level is significantly increased in Basal‐like TNBC (n = 243), p < 0.0001. (F) Representative phase contrast images of wound healing assay are shown, the white lines denote the width of the wound. (G) Representative images of spheres generated by SCR, KD31, KD32 were shown, scale bars 50 µm. (H) Representative images of spheres generated by MCF‐7‐Vector and MCF‐7‐serglycin were shown, scale bars 50 µm [file CTM2-11-e311-s002.tif]

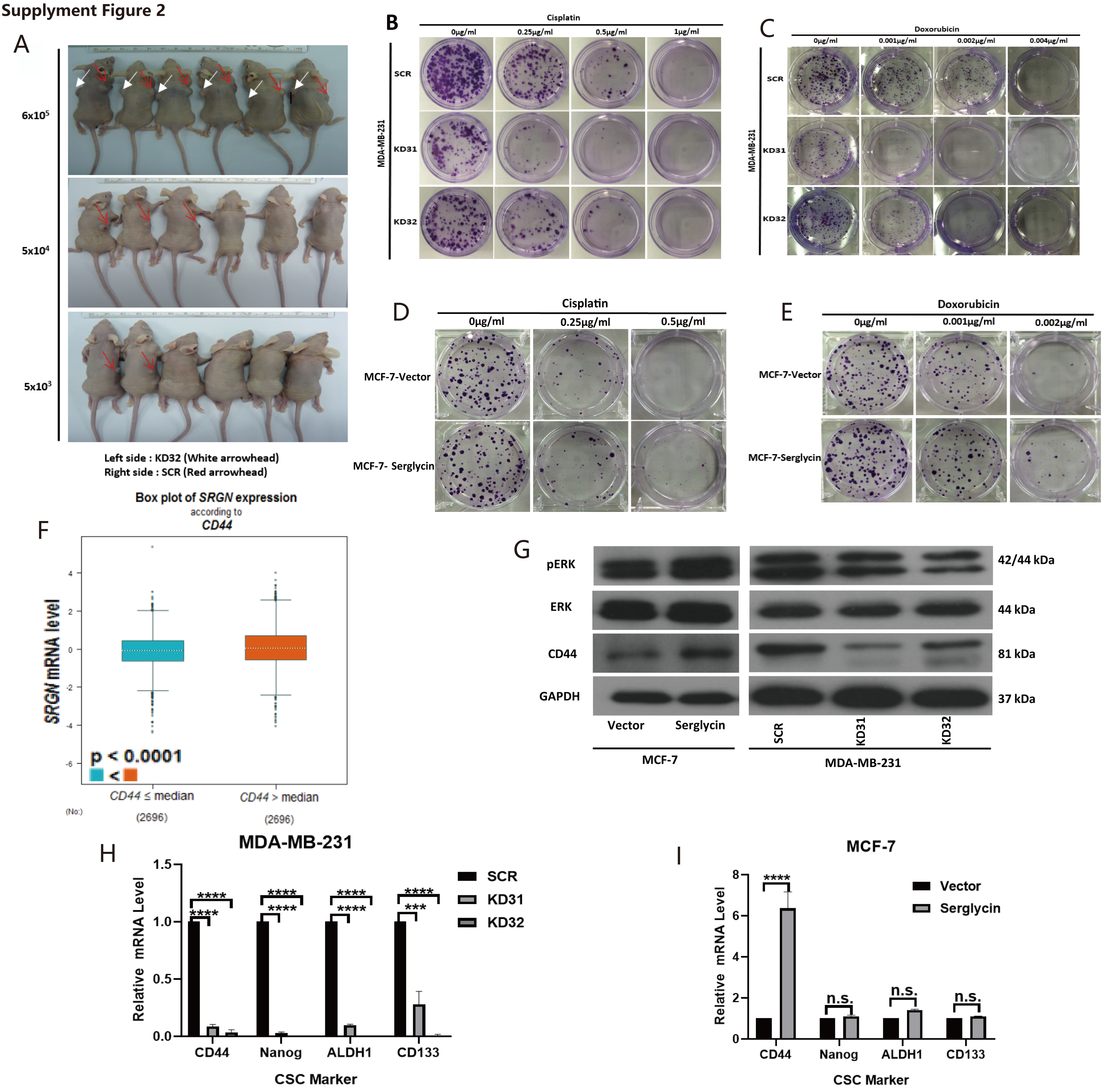

Supplement: Supplementary file 4 — Figure S2 (A) Tumor formation in nude mice when SCR or KD32 cells were injected subcutaneously into right or left armpit. (B and C) Representative images of colonies generated by SCR, KD31, and KD32, treated with different concentration of Cisplatin or Doxorubicin were shown. (D and E) Representative images of colonies generated by MCF‐7‐Vector and MCF‐7‐serglycin, treated with different concentration of Cisplatin or Doxorubicin were shown. (F) SRGN mRNA level is positively correlated to CD44, p < 0.0001 based on the bc‐GenExMiner v4.1 databases. (G) The protein level of CD44 and p‐ERK in stably transfected cells were analyzed by Western Blotting. (H and I) CSC markers such as CD44, Nanog, ALDH1, and CD133 mRNA expression in MDA‐MB‐132 and MCF‐7 by quantitative real‐time PCR. Data were presented as the mean ± SD of three independent experiments. *p < 0.05, **p < 0.01, ***p < 0.001 by student's t‐test [file CTM2-11-e311-s003.tif]

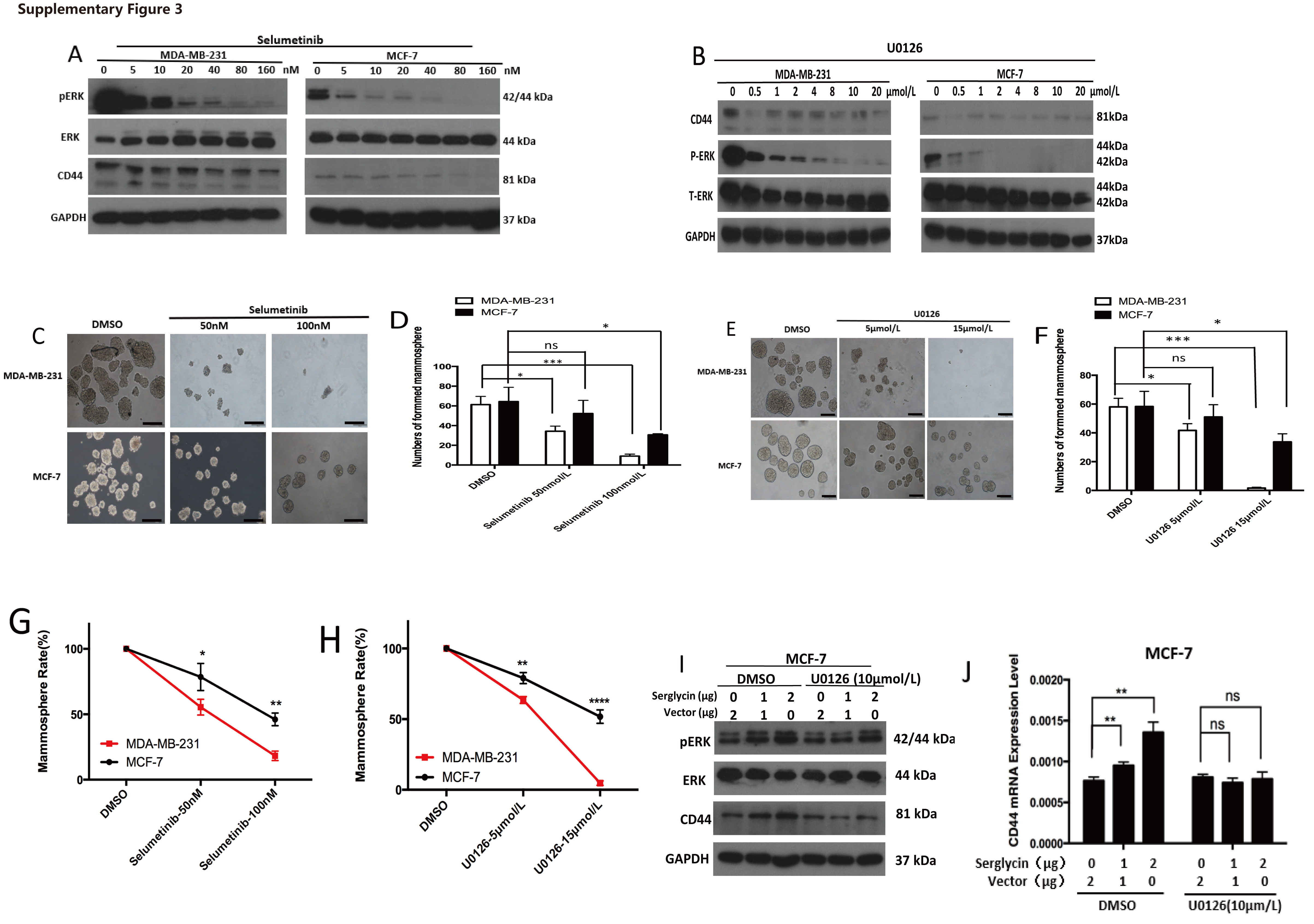

Supplement: Supplementary file 5 — Figure S3 (A and B) The protein level change of CD44 and p‐ERK in two wild type cells treated with different concentration of Selumentinib or U0126 (ERK inhibitor) was presented. (C and D) Representative images and bar graph of tumor spheres formed by two wild type cells, treated with increased dose of Selumetinib were shown, scale bars 50μm. (E and F) Representative images and bar graph of tumor spheres formed by two wild type cells, treated with increased dose of U0126 were shown, scale bars 50μm. (G and H) Numbers of mammospheres were counted after being treated with Selumetinib or U0126. (I and J) MCF‐7 cells treated with U0126 were transiently transfected with serglycin plasmid. The mRNA level of CD44 was determined by quantitative real‐time PCR (J). The protein level of CD44 and p‐ERK was analyzed by Western Blotting (I). [file CTM2-11-e311-s004.tif]

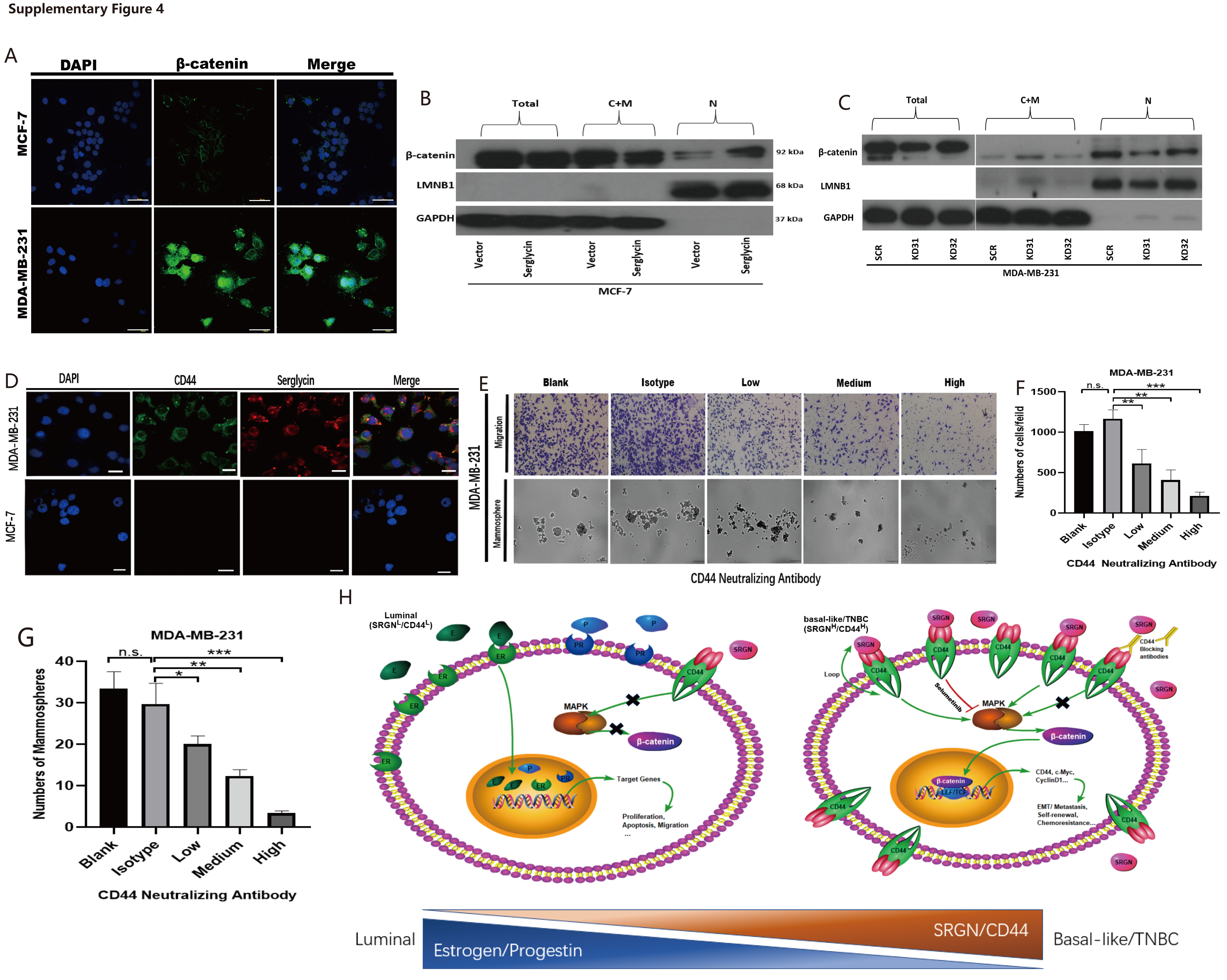

Supplement: Supplementary file 6 — Figure S4 (A) The expression level and the localization of β‐catenin in MDA‐MB‐231 and MCF‐7 were confirmed by confocal immunofluorescence, scale bars 50 µm. (B and C) Nuclear (N) and cytosolic/membrane (C+M) protein from stably overexpression cells (B) or stably knockdown cells (C) were subjected to examined β‐catenin change by Western Blotting. LMNB1 and GAPDH were used as loading control of N and C+M, respectively. (D) Immunofluorescence assay had also shown that SRGN and CD44 were colocalization on the membrane of MDA‐MB‐231, scale bars 20μm. (E) The ability of migration and self‐renewal of MDA‐MB‐231 cells can be prevented by CD44 neutralizing antibody in dose‐dependent. (F) The migration numbers of crossed cells counted by ImageJ are shown (from triplicates). (G) Numbers of mammospheres which represented self‐renewal of MDA‐MB‐231 cells were counted. (H) The CD44 neutralizing antibody is competitive inhibiting SRGN binding with CD44, meanwhile. Ultimately, the CD44 neutralizing antibody could inhibit the EMT, self‐renewal, and chemoresistanse of MDA‐MB‐231 by the MAPK pathway. [file CTM2-11-e311-s005.tif]
